# The Links between Human Diets and Health and Climate Outcomes in the World’s Macro-Regions during the Last 50 Years

**DOI:** 10.3390/ijerph17041219

**Published:** 2020-02-13

**Authors:** Christian Fischer, Pier Paolo Miglietta

**Affiliations:** Faculty of Science and Technology, Free University of Bozen-Bolzano, 39100 Bolzano, Italy; pierpaolo.miglietta@unibz.it

**Keywords:** sustainable diets, human health, world climate, food pessimism

## Abstract

Globally, traditional food security fears have been supplemented by concerns about food system sustainability that link current agricultural production practices to damages of environmental ecosystems and the world’s climate, thus threatening the natural resource base of future generations. This paper aims at creating a better understanding of the evolution of diet sustainability from 1961 to 2013. Data from the Food and Agriculture Organization of the United Nations were used to investigate the situation for the world as a whole as well as for its macro-regions Africa, Asia, the Americas, Europe and Oceania. We define diet sustainability by (a) the share of daily per capita calorie intake derived from vegetable/plant products and (b) the variety of vegetable/plant products consumed, measured by the Simpson diversity index. Moreover, total calorie consumption is considered. Then the correlations between diet sustainability and (a) macro-regional life expectancy rates and (b) food system greenhouse gas emissions are calculated. The results show that diet sustainability has not changed much during the last 50 years. Moreover, the nexus between diets and health and climate outcomes is not fully evident at the macro-regional level. Therefore, Malthus 2.0, i.e., scientific food pessimism, should be avoided. In particular, the limitations of dietary contributions to human and planetary health ought to be more widely acknowledged.

## 1. Introduction

Planet Earth’s natural resources, such as land, freshwater, raw materials etc., have to be shared among an increasing number of people (and farm animals). At the same time, human life expectancy has continuously increased [1]. Consequently, more people consume for longer, leading to fears whether there is enough for all of them, now and in the future.

One particular concern is about human diets and their resource implications. Globally, agriculture already accounts for up to 40% of global land use, 70% of freshwater withdrawals and 30% of greenhouse gas emissions [2]. The question is whether humankind can go on consuming food and drink as it currently does.

While food security fears have been with humans for their entire existence, the British scholar Thomas Malthus, at the turn of the 18th century, introduced the notion of the “population trap”. Rapid population growth would eventually outstrip agricultural production, thus leading to shortages of food supply and starvation [3]. 

Today, there are various other reasons for “pessimism on the food front”, the most important ones being [4]: (i) the limited availability of arable land; (ii) soil erosion and degradation; (iii) declining biodiversity with species losses such as insects in general and pollinators in particular; (iv) the increased use of agro-chemicals such as mineral fertilizers and pesticides, potentially leading to ‘environmental toxification’; (v) stagnating crop yields; (vi) declining wild fish and seafood stocks; and ever more pressing, (vii) the various threats resulting from global warming such as land losses caused by rising sea levels, crop and catch failures due to higher temperatures, changed moisture regimes and air composition, ocean acidification and natural disasters.

In short, besides the more traditional food security fears, many more recent concerns about food system sustainability have emerged which link current agricultural production practices to damages of environmental ecosystems and world climate, thus threatening the natural resource base of future generations. Hence, the current perception is that the very activity of food production that is meant to keep humans alive will kill them eventually in the long run.

According to the Intergovernmental Panel on Climate Change (IPCC), net anthropogenic greenhouse gas (GHG) emissions of the global food system (i.e., those from human activities) accounted for 21%–37% of worldwide total GHG emissions during 2007–2016 [5]. The global food system comprises all activities related to the agricultural/forestry/fisheries production, processing, distribution, preparation and consumption of food. Hence, total emissions include those from food production including land use changes (i.e., deforestation and peatland degradation) plus those from all pre- and post-production activities. Emissions from farms and agricultural land expansion represent 16%–27% of total anthropogenic food system emissions with livestock kept on managed pastures and rangelands accounting alone for about 10% of the global emissions’ total (ibid.). Therefore, IPCC argues that better balanced diets with higher intakes of plant-based foods, such as those based on coarse grains, legumes, fruits and vegetables, nuts and seeds, and animal-sourced food produced in resilient, sustainable and low-GHG emission systems, would represent major opportunities for global warming adaptation and mitigation (ibid.).

At the same time, diet-related health effects are ever more radically communicated. For instance, the EAT-Lancet study [6] (p. 447) states: “Unhealthy diets pose a greater risk to morbidity and mortality than does unsafe sex, and alcohol, drug, and tobacco use combined”. However, another even more recent and more comprehensive nutrition and health study comes to a more cautious conclusion [7] (p. 12): “In summary, we found that poor dietary habits are associated with a range of chronic diseases and can potentially be a major contributor to NCD (non-communicable disease) mortality in all countries worldwide.” Hence, parts of the mainstream nutrition and medical science communities proclaim diets to be major contributor to bad health and early death.

The definition of diet sustainability is complex since it comprises multiple criteria. One of the most comprehensive definitions of sustainable diets is provided by FAO/Biodiversity International [8] (p. 10):

*Sustainable diets are those diets with low environmental impacts, which contribute to food and nutrition security and to healthy life for present and future generations. Sustainable diets are protective and respectful of biodiversity and ecosystems, culturally acceptable, accessible, economically fair and affordable; nutritionally adequate, safe and healthy; while optimizing natural and human resources.*

This definition includes environmental, social and economic dimensions. However, given its broad and unspecific approach, the definition is difficult to use as a practical measurement concept. 

A systematic review of the measurement of sustainable diets was undertaken by Jones et al. [9]. The results show that GHG emissions and land use are some of the most commonly used metrics. Röös et al. [10] update the review and expand the discussion on suitable indicators for the environmental impact of human diets. While these studies propose a variety of measures related to climate change, land use, water use, energy use, biogeochemical flows and biodiversity, there seems to be general agreement that a low consumption of animal products is considered to have minimum negative environmental and climate consequences [5,11]. 

Rearing animals as a source of food requires large amounts of space and water for keeping them and for growing their feed [12]. The feed-to-food energy conversion ratio is small, meaning that significantly more feed calories are required to produce one calorie of animal-derived food product. However, this disregards the fact that most animal-derived food products are mostly sources of protein and particular vitamins (e.g., of the B group) and minerals (calcium, iron) rather than of nutritional energy. Moreover, in particular ruminants produce large amounts of GHG emissions (mostly methane during feed digestion) and therefore are seen as a major cause of global warming [13]. Finally, in particular large-scale or “industrial” animal rearing with large overall numbers and high animal-per-space unit ratios are often seen as unethical and a source of animal welfare problems.

Government recommendations on sustainable dietary guidelines from several countries are summarized by Gonzales-Fischer and Garnett [8]. They show that dietary diversity is a key recommendation for healthy food intake. This is also supported by the recommendations from the World Health Organization [14] and the EAT Lancet Commission [6].

Dietary diversity is beneficial because consuming a wide variety of food increases the likelihood to satisfy a broad spectrum of nutrient needs in a context where a single food contains only a limited range of nutrients [15]. Hence, consuming only a small selection of foods may provide oversupply of some nutrients and deficiencies of others. Moreover, dietary diversity decreases the probability of over-consuming potentially contaminated food items, therefore limiting the risk of foodborne illnesses. However, some recent study evidence suggests that consuming a large variety of foods may also lead to including a larger number of highly processed products, potentially leading to suboptimal eating patterns and weight gain in adults [16]. 

While dietary diversity is a commonly used concept (e.g., [17]), variety in food intake can occur in several ways. First, regarding the type of food consumed, dietary diversity would require that many different foods be consumed, i.e., different types of vegetables, cereals, dairy products etc. Second, regarding the source of food products, the same type of food may come from different origins (e.g., apples consumed may be produced in different countries). Third, regarding variety over time, diet diversity may be achieved over a longer period thus reflecting seasonal variations in food supply, however, with a limited range of food intake during single days, weeks or months. In the context of this study, dietary diversity refers to the first diversity concept, i.e., the number of different food items consumed during the entire period of one year.

This article aims at creating a better understanding of the evolution of aggregate diet sustainability during the last 50 years. Data from the Food and Agriculture Organization of the United Nations (FAOSTAT) were used to investigate the situation for the world as a whole as well as for its macro-regions Africa, Asia, the Americas, Europe and Oceania. Diet sustainability is defined using two indicators that reflect the supposed healthiness and climate impacts of the food consumed. At the same time, the total amount of calories that are consumed worldwide and in the individual macro-regions are considered. Then the correlations between diet sustainability and (a) macro-regional life expectancy and (b) food system GHG emissions are provided. After a discussion of the results, the paper concludes that diet sustainability has not changed significantly during the last 50 years for the world as a whole and in most of its macro-regions.

## 2. Methods and Data

At the macro-regional level, average diets are broad aggregates that reflect structural differences in the composition of calorie intakes across different but geographically similar countries. Such macro-level diets cannot account for intra-regional consumption heterogeneities, i.e., variations of diets within the macro-regions. However, such macro-regional diet indicators should be useful to track average diet changes within the same regions over time, which is the purpose of this study.

Thus, this study investigates the evolution of the world’s macro-regional diets between 1961 and 2013, using three indicators:

a) the share of daily per capita calorie intake derived from vegetable/plant products (range from 0 to 1);
b) the variety of vegetable/plant products consumed, measured by the Simpson diversity index [18] (range from 0 = no to 1 = maximum variety);
c) total annual food calories consumed in a given macro-region and year, reflecting the contributions of both total population in this area and its per capita calorie intake per day.

The Simpson or Berry Index is a diversity measure that takes into account the evenness of calorie consumption or, in other words, the relative distribution of calories across individual foods as well as the total number of products consumed (i.e., consumption richness). The index measures whether the nutritional energy consumed is derived more or less equally from a large number of food (high diversity) as opposed to a situation where only a few foods contribute most of the energy intake (low diversity) [16]. This measure has been used in economic food diversity studies [15] and is conceptually similar to the Herfindahl or Herfindahl–Hirschman index in economics or the Gibbs–Martin index in sociology, psychology and management studies. 

We calculate diet diversity (*D*) as follows:

$$D=1-\left(\frac{\sum n\left(n-1\right)}{N\left(N-1\right)}\right)$$

where *n* is the number of calories consumed in a particular year derived from a particular plant product and *N* is the total number of plant products consumed in one year (richness).

The years covered are: 1961, 1970, 1980, 1990, 2000, 2010, and 2013. The raw data were taken from FAOSTAT, Food Balance Sheets, food supply (kcal/capita/day).

Food balance sheet supply data can be regarded as a useful and valid proxy measure for human per capita consumption. They reflect the total supply of a food item in a country (production + imports − exports + changes in stocks) available for human consumption in a particular year, divided by the total population of that country in that year [19]. However, it is a calculated average indicator rather than one that is actually measured at individual or household level.

In general, FAO data are used as decision basis for policies in agriculture and food by governments and organizations around the world. FAO data collection is subject to quality control and the published data fulfill the commonly required quality criteria such as relevance, accuracy and reliability, timeliness and punctuality, coherence and comparability, and accessibility and clarity [20]. However, using data in particular from developing countries and from as far back as 1961 involves risks since the analysis results can only be as good as the used data. Nevertheless, these data are used widely by national and international organizations, including IPCC. 

## 3. Results and Discussion

This section presents the results in three parts. First, the summary of the state of the world’s average diet and the evolutions of the macro-regional diets are presented and discussed. Then the situation in the different macro-regions as well as the one of the world’s and the decade-by-decade change in the average diets are provided. Finally, we present and discuss the results from the correlation analysis between the different sustainability and health indicators.

### 3.1. Summary of the World’s Sustainable Diet Situation

**Figure 1:**
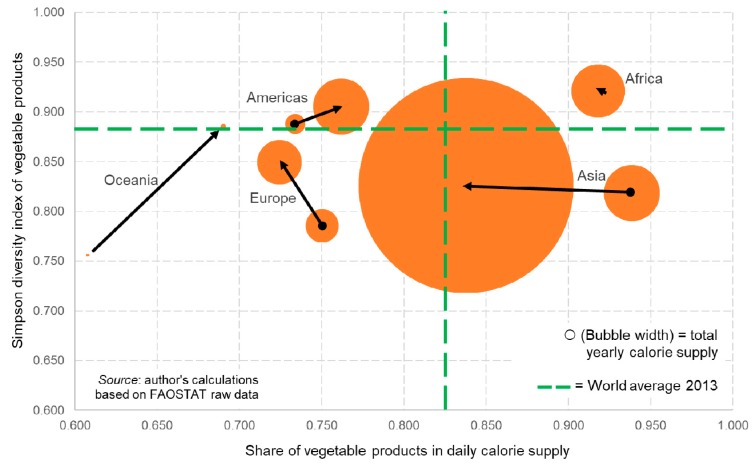
World diet situation, by macro-region, 1961 and 2013.

The world average diet became more based on animal products and remained all but unchanged regarding the variety of vegetable products consumed (Figure 1). The share of kcal in daily food intake derived from vegetable products decreased from 84.6% (1961) to 82.2% (2013), while plant product variety decreased slightly from a Simpson index score of 0.888 to 0.881. Both changes are small. During the same period, total world calorie supply and therefore approximate consumption more than tripled (from 2.48 × 10^15^ kcal per year to 7.59 × 10^15^ kcal per year; not shown in Figure 1). 

Macro-regional consumption patterns and trends are not uniform (see Figure 1). In Africa, the share of vegetable products in the regional diet slightly fell from 92.2% (1961) to 91.8% (2013) while the variety index score remained virtually unchanged (0.920 to 0.921). Over the same period, total calories supplied increased more than fivefold. The diet of the Americas improved in both measures (vegetable share 73.4% to 76.2%, plant product variety from 0.888 to 0.905), while total calorie supply almost tripled. In Asia, the share of vegetable products in the diet fell from 93.9% to 83.8%, the variety index score slightly improved from 0.819 to 0.826 and total calories supplied almost quadrupled. The European diet’s share of vegetable products decreased from 75.0% to 72.4%, while vegetable product variety increased from 0.786 to 0.849, and total calorie supply increased by 34%. Finally, the Oceanian diet also improved in both measures (plant-product share from 60.8% to 69.0%, plant product variety from 0.756 to 0.885) while total calorie supply increased by a factor of more than 2.5.

### 3.2. Evolution of Macro-Regional Diets

**Figure 2:**
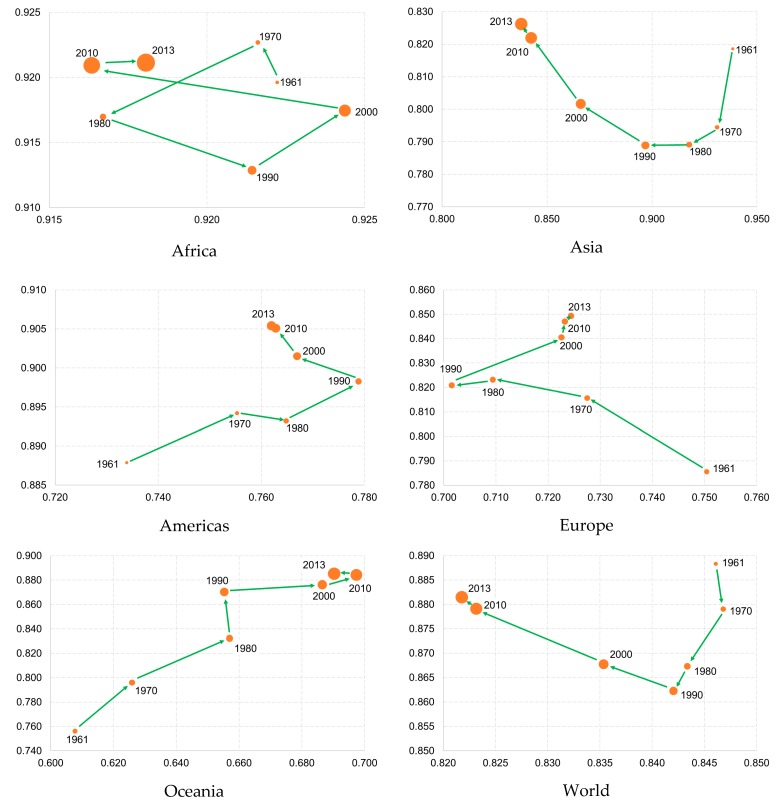
Evolution of the diet situation by macro-region and for the world as a whole, 1961 to 2013. In all charts: *y*-axis: Simpson’s index of diversity of vegetable products; *x*-axis: share of vegetable products in total calorie supply (%); bubble width = total calorie supply (kcal/year).

The evolution paths of diet sustainability for each macro-region are depicted in Figure 2. As before, it shows the share of calories deriving from plant products on the *x*-axis, the diversity within the plant calories consumed on the *y*-axis and the total amount of calories supplied/consumed as the size of the bubble for each year. 

The African diet evolved without a clear trend and rather unsystematically which may reflect potential data reliability problems within this macro-region. Overall, the plant-calorie share decreased by 0.5% over the observed 52-year period while the diversity index increased by 0.2%.

The Asian diet evolution displays a distinct *U* curve pattern. While the share of calories deriving from plant products continuously decreased, plant-diet diversity decreased from 1961 to a low of 0.789 in 1990 and then it increased again steadily until 2013. The total changes over the 52 years are −10.7% for the plant-calorie share and 0.9% for the diversity index.

The diet of the Americas displays a continuous improvement in both measures of sustainability until 1990. After that, the calories derived from plant products (77.9% in 1990) decreased again to 76.2% in 2013, resulting in an overall increase of 3.8% over the 52 years. At the same time, diet diversity steadily improved with a total increase of 2.0%. 

The European diet continuously became less plant calorie-based from 1961 to 1990 (70.2%) and then increased again steadily in 2013. Overall, this measure decreased by 3.5% over the observed 52-year period. During the same time, the diversity index increased by 8.1%.

The diet in Oceania, as in the Americas, shows a clear trend of improvement in both measures. The calorie share derived from plant products improved by 13.6% from 1961 to 2013. Over the same period, dietary diversity increased continuously by 17.1% overall. 

Finally, the evolution of the world diet displays a decrease of both measures from 1961 to 1990. After this year, the plant-product share continues to decrease while the dietary diversity score continuously improved again. The total changes during the 52-year period are −2.9% for the plant-calorie share and −0.8% for the diversity index. 

### 3.3. Links between Diets and Health and Climate Indicators

Life expectancy (at birth) rates improved in all macro-regions over the period analyzed. According to Riley [1] and the Institute for Health Metrics and Evaluation [21], the world average lifespan increased by 48% from 48 years (1950) to 70.8 years (2013). In Africa, over the same period, it increased by 65% to 58.8 years, in the Americas by 31% to 76.5 years, in Asia by 73% to 71.8 years, in Europe by 25% to 80.6 years, and in Oceania by 22% to 77.5 years. 

Life expectancy and disability depend on multiple factors. The leading causes for death in 2017 were ischemic heart disease, neonatal disorders, stroke, lower respiratory infections, diarrhea, road injuries and chronic obstructive pulmonary disease [21]. The leading causes for disability in 2017 were low-back pain, headache disorders, depressive disorders, diabetes, age-related hearing loss and chronic obstructive pulmonary disease (ibid.). Suboptimal diets only account for about 22% of deaths and 15% of all disabilities globally [7]. Therefore, it can be expected that the correlations between the macro-regional changes in diet sustainability and the corresponding life expectancy rates are weak.

Table 1 provides the Pearson correlation coefficients between the macro-regional life expectancies and the diversity of plant-product index scores as well as between life expectancies and the shares of energy intake derived from plant products. Hence, two correlation coefficients are calculated between the data for the macro-regions in 2013, thus indicating cross-sectional association of whether macro-regions with higher scores in the diet sustainably measures show higher life expectancy rates. In addition, another two correlation coefficients are calculated between the overall change rates from 1961–2013 for the two diet sustainability measures and the overall change rate from 1950–2013 for the life expectancy numbers. These correlation coefficients indicate the association between the improvement rates, i.e., whether, for instance, macro-regions that improved more in diet sustainability also improved more in life expectancy. 

The links between diet diversity and life expectancy as well as between plant-product content and life expectancy are contrary to expectations both for the absolute values and for changes over time. According to Table 1, correlation coefficients are negative and larger for plant-product content (−0.9 and −0.7) than for diet diversity (−0.5 and −0.7). This confirms that the links between diet and health outcome are weak as indicated above. 

Health determinants comprise lifestyle factors (diet, exercise, sleep, drug use, social contact etc.), biological and genetic factors (age, inherited risks etc.), environmental factors (exposure to pollution, noise, radiation etc.) and, finally yet importantly, also luck (avoidances of accidents, violence etc.) [22,23]. Hence, human mortality as well as morbidity is influenced by much more than diets and therefore even optimal food intake cannot guarantee healthy lives.

As mentioned above, food systems contribute 21%–37% of global anthropogenic GHG emissions of which agricultural production, including indirect emissions resulting from land-cover change, contribute 52%–86% of total food system emissions (see also [24,25]). In 2013, food represents 97% of world agricultural production (in value/dollar terms) and 23% of world food production was exported, according to FAOSTAT Production and Trade databases (online).

Regionally, agricultural production can differ from food consumption. In 2013, the shares of food in agricultural production value were: Africa 98%, Americas 97%, Asia 96%, Europe 99.5% and Oceania 85%. In the same year, net food exports represented −11% of production in Africa, 12% in the Americas, −5% in Asia, −2% in Europe and 50% in Oceania. For 1961, no comparable data are available in the FAOSTAT Production and Trade databases (online).

Macro-regional per capita food system GHG emissions in 2013 amounted to (kg CO^2^eq): Oceania 1,939, Americas 1,153, Africa 816, Europe 779 and Asia 527. These values are agricultural production emissions from the FAOSTAT Emissions database (online) adjusted for food shares and net exports.

The correlation coefficient between 2013 macro-regional diets’ plant-product shares and food system per capita GHG emissions is −0.6 (Table 1). Europe, in particular, does not fit the pattern since it has low emissions despite a low plant-product share. Nevertheless, the correlation is negative as expected: the higher in a macro-regional diet the share of the calories consumed that are derived from plant products the lower the per capita food system GHG emissions are in this macro-region. 

However, it should also be mentioned that world agricultural per capita GHG emissions fell by 19% from 1961 (890 kg CO^2^eq) to 2013 (719 kg CO^2^eq) according to the FAOSTAT Emissions database (online).

## 4. Conclusions

Assuming data reliability and method validity, the presented analysis suggests that the sustainability of diets at the world and macro-regional levels has not changed much during the last 50 years. For the world as a whole, the share of calories consumed derived from plant products decreased by −2.9% and the plant-product dietary diversity index score decreased slightly by 0.8%. Macro-regionally, the diets of the Americas and Oceania improved in both measures from 1961 to 2013, the African diet remained largely unchanged, and the European and Asian diets became more animal-calorie based while they improved in plant-product diversity (Europe) or remained virtually unchanged in this regard (Asia). 

Moreover, the nexus between diets and health, and diets and climate outcomes is not fully evident at the macro-regional level. The correlations between macro-regional life expectancy and diet sustainability are negative and therefore contrary expectations. This confirms that the link between human diets and overall health is weak since human mortality and morbidity have multiple, non-diet related causes. In particular, improvements in the medical systems and medicinal availability have contributed to longer and healthier lives—factors that are unrelated to food intake.

However, as mentioned above, during the analyzed 52-year period total world calorie consumption more than tripled while global population only increased by a factor 2.3. As a result, in 2018, 39% of the world adult population is considered overweight and another 13% obese [5]. This relative recent malnutrition phenomenon and its health consequences are very likely not yet reflected in the current life expectancy data.

At the same time, our analysis shows that there is a negative correlation between a macro-regional diet’s plant-product calorie share and its per capita food system GHG emissions. However, at 0.6 the correlation is not strong and in particular Europe has low emissions despite a relatively higher share of animal-originated calories in its diet. Moreover, global agricultural per capital GHG emissions fell by almost 20% between 1961 and 2013. 

In the future, food system GHG emissions are probably better tackled at the supply side by tightening production standards in particular for ruminant products, which would cause their costs and prices to rise and their demand and consumption to fall. This could be achieved by new and stringent regulations, for instance regarding the type of used animal feeds, feeding and manure management practices, and mandatory feeding additives the use of which would result in lower livestock GHG emissions (supplement to [5]).

More generally, market-based solutions such the global introduction of carbon pricing as either carbon taxation, or emission trading (cap and trade, or CAT) schemes, would be most efficient and effective even if less popular [26]. Such schemes would make the production and distribution and hence the consumption of products with heavy GHG emission footprints more expensive and therefore limit or abolish them altogether.

In any case, Malthus 2.0, i.e., scientific food pessimism should be avoided [27]. A first step in this direction would be a wider acknowledgement of the limitations of dietary contributions to human and planetary health.

## Figures and Tables

**Table 1:** Pearson correlation coefficients between various measures of diet sustainability, life expectancy and GHG food system emissions.

	Diversity of Plant Products Index 2013	Change (1961–2013) of Diversity of Plant Products	Share of Energy Intake Deriving from Plant Products 2013	Change (1961–2013) of Energy Intake Deriving from Plant Products
**Life expectancy (at birth) 2013**	−0.478	--	−0.929	--
**Change (1950–2013) of life expectancy**	--	−0.714	--	−0.696
**Food system GHG emissions per capita 2013**	--	--	–0.640	--

Number of observations for each correlation: five macro-regions, representing the entire world.
